# Efficacy and Safety of Intelligent Microfocused Ultrasound in Lifting Facial Laxity: A Multicenter, Prospective, Randomized, Split‐Face Study

**DOI:** 10.1111/jocd.71106

**Published:** 2026-08-19

**Authors:** Shuzhan Shen, Jingjing Li, Qing Fan, Haiyan Zhang, Minzhi Wu, XueLei Li, Shuping Guo, Qian Yang, Chenchen Wang, Ling Ma, TianHua Xu, Xiuli Wang, Peiru Wang

**Affiliations:** ^1^ Institute of Photomedicine Shanghai Skin Disease Hospital, School of Medicine, Tongji University Shanghai PR China; ^2^ Department of Dermatology The Fifth People's Hospital of Suzhou Suzhou PR China; ^3^ Department of Dermatology Shanghai Fengxian District Central Hospital Shanghai PR China; ^4^ Department of Plastic and Reconstructive Surgery Changzhi People's Hospital Changzhi PR China; ^5^ First Hospital of Shanxi Medical University Taiyuan PR China; ^6^ XEMIS Medical Technologies Limited Shenzhen PR China; ^7^ Department of Medical Aesthetics Qilu Hospital of Shandong University Jinan PR China; ^8^ Department of Cosmetic Medicine Shenzhen Nanshan People's Hospital Shenzhen PR China

## Abstract

**Background:**

Age‐related dermal collagen depletion compromises skin elasticity, manifesting as tissue laxity and loss of facial structural definition. The pursuit of facial tightening has driven vigorous development of non‐surgical rejuvenation modalities, with microfocused ultrasound emerging as a clinically validated intervention for restoring cutaneous firmness.

**Objectives:**

This study aimed to assess the efficacy and safety of novel intelligent microfocused ultrasound (iMFU) in reversing facial aging and improving skin sagging.

**Methods:**

Participants with facial aging and sagging were enrolled and iMFU treatment was performed on the randomized middle and lower hemiface. The length, angle, and area of the nasolabial folds and perioral wrinkles, the angle of the side face and the lower face, melanin index (MI), and erythema index (EI) were recorded and evaluated at 1st and 2nd month post‐treatment. Side effects and subjects' satisfaction scores were evaluated during treatment and each follow‐up visit.

**Results:**

32 patients successfully completed the study. Quantitative analysis revealed obvious changes following iMFU treatment: the average length of perioral wrinkles and nasolabial folds on iMFU sides decreased by 7.2% (*p* < 0.001) and 18.5% (*p* = 0.011) in month‐2 follow‐up time. IMFU treatment also showed significant improvements in nasolabial fold angle (from 29.5 ± 4.3 to 30.7 ± 2.1) and area (37.5 ± 0.6 to 35.1 ± 1.2). Perioral folds achieved a similar therapeutic effect. Side face angle a demonstrated a significant increase from 12° ± 2.6° to 13° ± 1.5°, while the mandibular angle b similarly increased from 16° ± 2.4° to 18° ± 1.9° on iMFU sides. In addition, iMFU sides had greater benefits in improving MI and EI than the control sides. The subjects' satisfaction scores in iMFU sides were significantly higher than that in control sides. Except for 1 patient who experienced toothache after iMFU treatment, no persistent or serious side effects occurred in the other subjects.

**Conclusion:**

IMFU treatment demonstrates both safety and efficacy for facial rejuvenation, with clinically validated outcomes in skin tightening, facial contour refinement, and skin tone improvement. This non‐invasive approach delivers significant aesthetic enhancement across multiple parameters of facial aging.

## 1 Introduction

Progressive aging leads to accelerated collagen depletion, diminished skin elasticity, and gravitational descent of facial fat pads, collectively undermining facial definition [1, 2]. Concurrently with rising living standards, public awareness of facial skin laxity has heightened considerably, fueling increasing demand for facial rejuvenation therapies [3, 4].

Surgical intervention persists as the principal therapeutic modality for skin laxity recently. Inherent limitations, including its invasive nature and protracted recovery requirements, have instigated a paradigm shift toward less invasive alternatives [4, 5]. Concurrently, technological innovation and escalating patient demand for safe, efficacious treatments with expedited recovery profiles have established non‐invasive skin tightening as a central research priority within dermatology [6]. This evolution has precipitated accelerated advancement in facial rejuvenation technologies in recent years, characterized by both novel device development and emergent pharmacological agents.

Contemporary non‐surgical interventions are classified into three principal modalities: physical, chemical, and biological approaches [7, 8]. Energy‐based devices, specifically lasers, radiofrequency systems, and focused ultrasound technology, have gradually become cornerstone therapies for skin rejuvenation. These techniques exhibit distinct advantages characterized by minimal invasiveness, abbreviated recovery periods, and demonstrable clinical efficacy. Their underlying therapeutic mechanisms collectively confer multifunctional benefits, including reduction of wrinkles, refinement of skin texture, clearance of dyspigmentation, and resolution of telangiectasias [9, 10].

Microfocused ultrasound, a non‐surgical aesthetic modality, employs precisely targeted ultrasonic energy to selectively engage the deep dermis and subcutaneous fascial layer [11, 12]. This generates controlled molecular oscillation and localized thermal coagulation points within treated tissues, thereby stimulating immediate collagen contraction and initiating neocollagenesis. The resultant tissue remodeling effectively restores cutaneous elasticity, tightens lax skin, attenuates wrinkles, and recontours facial architecture. Consequently, this technology has gained widespread international adoption for the clinical management of facial skin laxity [13, 14, 15, 16].

The novel intelligent microfocused ultrasound (iMFU) employed in our study enables precise energy delivery to the superficial musculoaponeurotic system (SMAS) and subcutaneous adipose layer, creating controlled thermal injury zones (TIZ) within targeted areas. This process facilitates collagen denaturation and contraction while maintaining the integrity of superficial and surrounding tissues. IMFU incorporates additional technological features relative to conventional MFU platforms, including an integrated real‐time ultrasound diagnostic module and adaptive variable focal‐length transducers (T ∼ and S ∼ probes) capable of dynamically adjusting treatment depth to 3.5–5.5 mm based on individualized SMAS topography. The potential clinical implications of these features warrant prospective evaluation [17, 18, 19, 20].

The 633 nm red light wavelength elicits specific biological effects on intracellular chromophores and mitochondria, demonstrating therapeutic benefits including accelerated wound healing, anti‐inflammatory effects, relief of muscular fatigue, and enhanced tissue repair [21, 22]. Consequently, this investigation implemented 633 nm red light as an adjunct therapeutic modality immediately following focused ultrasound treatment to relieve the discomfort after iMFU treatment. This protocol was designed to accelerate the resolution of transient iMFU‐induced inflammation through targeted modulation of mitochondrial cytochrome c oxidase activity and subsequent reduction of pro‐inflammatory cytokines.

Given the current paucity of domestic research on iMFU for facial skin laxity, this prospective investigation aims to validate the clinical efficacy and safety profile of iMFU in reversing facial skin aging. Therefore, we took the lead in uniting centers across China to jointly carry out the present study. This study not only evaluates the efficacy and safety of iMFU for reversing facial photoaging, but also provides evidence‐based support for clinical management of skin aging.

## 2 Method

### 2.1 Patient Selection

This study was designed as a multicenter, prospective, split‐face, observer‐blind investigation. 29 patients aged 40–65 years with facial soft tissue laxity who had a desire for rejuvenation treatment were recruited from multicenter between December 2024 and March 2025. The age, gender, treatment sessions, subjects' satisfaction, and pain evaluation were recorded. All subjects signed informed consent.

Exclusion criteria included patients with a history of keloid formation or hypertrophic scarring, facial cosmetic procedures (e.g., hyaluronic acid fillers, botulinum toxin injections) within the preceding 6 months, recent facial treatments (photoelectric therapies or topical regenerative medications) within 1 month, and severe systemic conditions including: chronic medical diseases, psychological/psychiatric illnesses, infectious diseases, and gestational or lactating status, or planned pregnancy during follow‐up.

All the clinical data conducted by each center were submitted to trained standardized evaluator evaluators for observation and analysis.

### 2.2 Treatment Protocol

**FIGURE 1:**
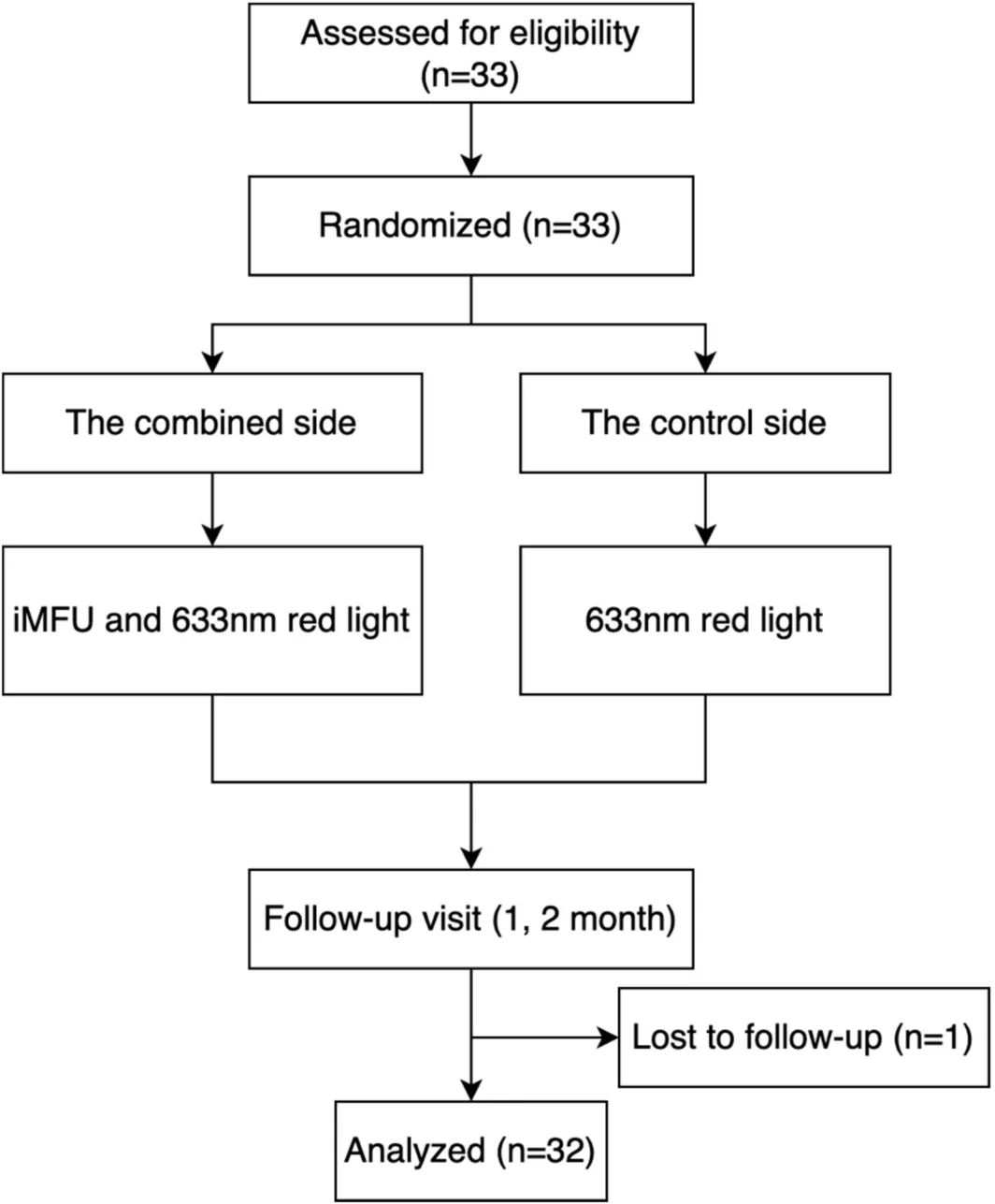
Flowchart of the study.

The treatment protocol was exhibited in Figure 1. The treated side and control side were determined using a random number table. All patients received iMFU treatment on one side of the face, followed by 633 nm red light irradiation on bilateral face. The treated side (iMFU+red light) received a combination of iMFU and 633 nm red light treatment, while the control side (red light) only received 633 nm red light treatment.

**FIGURE 2:**
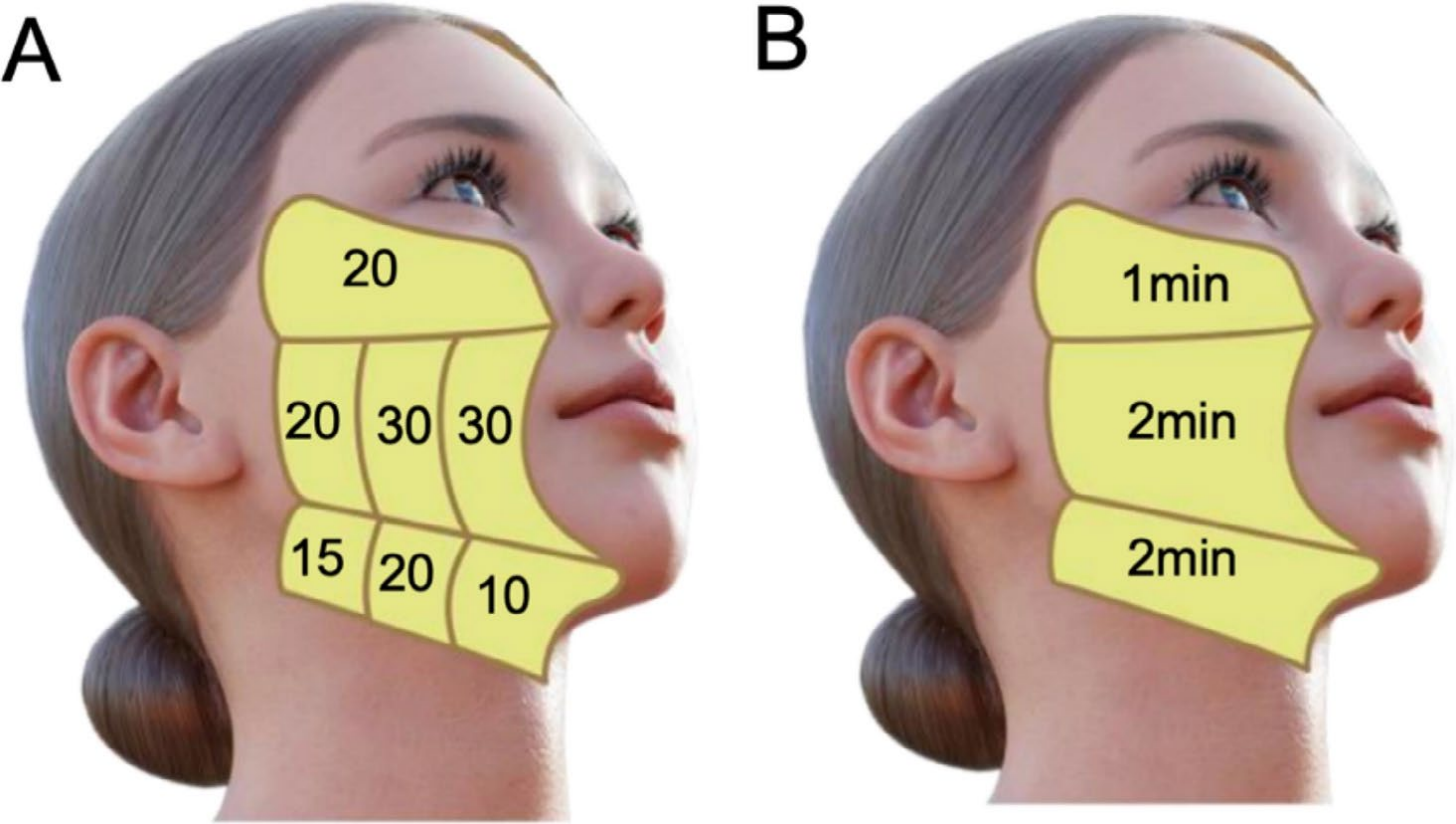
Recommended treatment protocol of iMFU. Transducer selection and energy delivery parameters were optimized according to facial topography. The linear handpiece (T ~ and T3.0 probe) (Figure 2A) was deployed with customized exposure line patterns, while the focal‐point handpiece (S ~ and S3.0 probe) (Figure 2B) was deployed with customized exposure duration. Both modalities utilized region‐specific treatment protocols based on anatomical landmarks.

Before treatment, the subjects' faces were washed with water. All study participants underwent treatment without topical anesthesia and the ultrasound coupling agent was evenly applied to the patient's facial area. IMFU was equipped with four distinct transducer configurations (T~, S~, T3.0, S3.0), and the specific parameters are dynamically adjusted according to the patient's tolerance level. Ultrasound measurement shows that the skin depth adjustment range accessible to the adaptive variable focal length depth sensor (T~and S~ probe) is 3.5–5.5 mm and traditional 3.0 mm focal length depth sensor (T3.0 and S3.0 probe) is 3.0 mm. IMFU transducers were uniformly configured with 25 mm focal depth at 1.5 mm spacing intervals. IMFU treating area extended from the lowest point of the inferior orbital margin to the horizontal line above the thyroid cartilage. An average treatment density of 300 linear passes and 10 000 focal points was administered to randomly assigned facial hemispheres in the lower two‐thirds, following manufacturer‐suggested treatment guidelines (Figure 2). This treatment protocol is indicated for patients presenting moderate facial adiposity or lean tissue volume. Treatment parameters require individualized calibration accounting for the subject's unique osseous structure, muscular tone, and adipose distribution. A personalized therapeutic design must be developed through comprehensive facial assessment, with energy delivery and transducer selection dynamically adjusted according to the patient's anatomical profile.

IMFU treatment was performed on the treated hemiface firstly, followed by 633 nm red light (XEMIS Medical Technology Co. LTD) treatment on the whole face. Follow‐up was conducted at 1 month and 2 months after treatment.

### 2.3 Assessment of Treatment Efficacy

**FIGURE 3:**
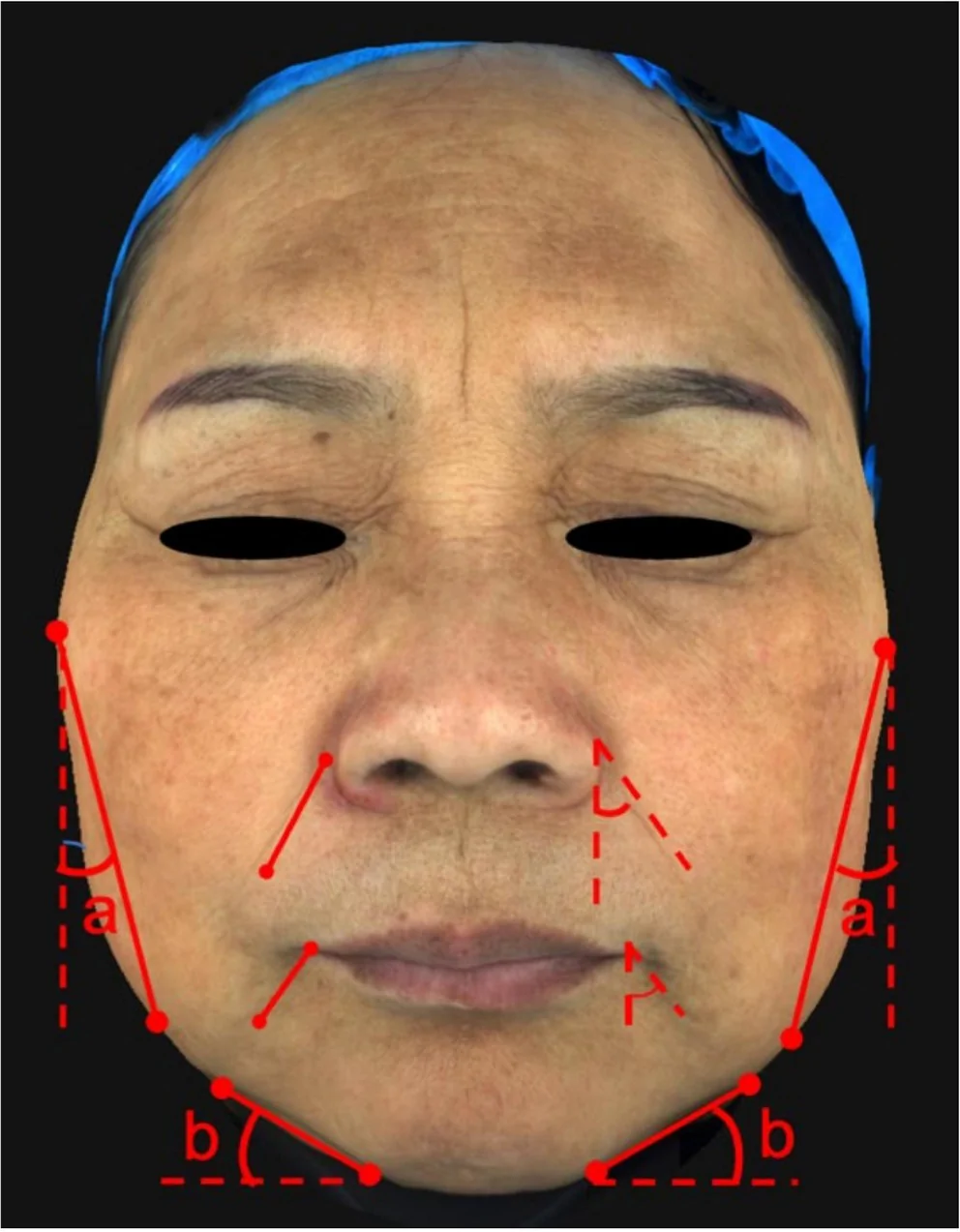
Representative photographs demonstrating the measurement protocols for nasolabial folds and perioral wrinkles and the angle of side face and lower face. For nasolabial fold length quantification, measurements were taken from the superior‐most point of the depression on one side to the corresponding inferior‐most point within the same shadowed fold region. Nasolabial fold angle was defined as the angular deviation between the fold's primary contour line and the vertical axis. Nasolabial fold surface area was automatically calculated using the integrated 3D facial imaging system employed in this study. Perioral wrinkle assessment followed identical measurement principles to those applied for nasolabial folds. Angle a is the angle between the tangent line of one side of the face (the line connecting the two most prominent points on the side face) and the vertical line, and angle b is the angle between the tangent line of the lower face (the line connecting the two most prominent points on the lower face) and the horizontal line.

Prior to treatment, immediately after treatment and 1 and 2 months after treatment, all subjects received photographic documentation via a facial 3D imaging system (Xitu‐4200, Xemis Medical Technology Co. LTD). Using Photoshop software (version 2020), the length, angle, and area of both nasolabial folds and perioral wrinkles, angles a and b were subsequently measured and evaluated (Figure 3). Additionally, the assessment parameters encompassed skin physiological measurements—specifically the melanin index (MI) and erythema index (EI)—which were also evaluated using the facial 3D imaging system.

Overall appearance and skin tone changes were assessed using the Global Aesthetic Improvement Scale (GAIS) at the 1‐month and 2‐month post‐treatment intervals. The GAIS employs a scoring range from −1 to 3, where: −1 indicates a worsened appearance compared to baseline; 0 signifies no significant change from baseline; 1 represents slight improvement; 2 denotes marked improvement; and 3 reflects optimal cosmetic outcomes. All evaluations were conducted by two independent dermatologists under standardized lighting and positioning conditions, performing assessments in a blinded manner.

Subjects' satisfaction was assessed bilaterally using an investigator‐developed questionnaire, where responses were scored as follows: 1 = unsatisfied, 2 = somewhat satisfied, 3 = mostly satisfied, and 4 = very satisfied. Concurrently, pain levels experienced during iMFU treatment were quantified using a Visual Analog Scale (VAS), while any additional adverse effects were systematically documented throughout the follow‐up period.

### 2.4 Study Outcomes

The primary efficacy endpoint comprised the length and angle of the nasolabial folds and perioral wrinkles, angle of side face and lower face. Secondary outcome included GAIS scores, MI and EI index, and subjects' satisfaction metrics.

### 2.5 Statistical Analysis

Statistical computations were conducted utilizing version 22.0 of SPSS (IBM Corp.). For comparative analysis of matched continuous variables demonstrating normal distribution, parametric paired *t*‐tests were employed. Nonparametric alternatives (Wilcoxon signed‐rank tests) were implemented when distributional assumptions were violated. Continuous variables conforming to normality assumptions were expressed as arithmetic means accompanied by standard deviations (SD), whereas skewed distributions were characterized using medians with interquartile ranges (IQR). *p* Values < 0.05 were considered to be statistically significant.

## 3 Results

From December 2024 and March 2025, a total of 33 patients were enrolled. 32 patients received complete treatment and went through the whole follow‐up visits, with the last patient finishing the follow‐up in May 2025. The study finally included 32 patients, aged from 39 to 59 years, with 30 females and 2 males. Before initiating the treatment procedure, baseline characteristics of both sides of the facial features were evaluated to be statistically similar (*p* > 0.05) (Table 1).

**TABLE 1:** Baseline characteristics of patients on iMFU+red light side and red light side.

Characteristic	Treatment		
	iMFU+red light Side, *N* = 29	red light side, *N* = 29	*p*
The length of nasolabial folds Mean ± SD (mm)	19.4 ± 3.6	20.4 ± 4.1	0.124
The angle of nasolabial folds Mean ± SD (°)	29.5 ± 4.3	28.9 ± 6.7	0.525
The area of nasolabial folds Mean ± SD (mm^2^)	37.5 ± 0.6	41.3 ± 0.8	0.381
The length of perioral wrinkles Mean ± SD (mm)	5.4 ± 2.1	4.6 ± 1.9	0.382
The angle of perioral wrinkles Mean ± SD (°)	39.6 ± 3.6	40.2 ± 2.8	0.185
The area of perioral wrinkles Mean ± SD (mm^2^)	7.8 ± 0.3	8.1 ± 0.2	0.162
Angle a Mean ± SD (°)	12 ± 2.6	12 ± 1.8	0.483
Angle b Mean ± SD (°)	16 ± 2.4	16 ± 1.9	0.327

### 3.1 Primary Outcomes

**FIGURE 4:**
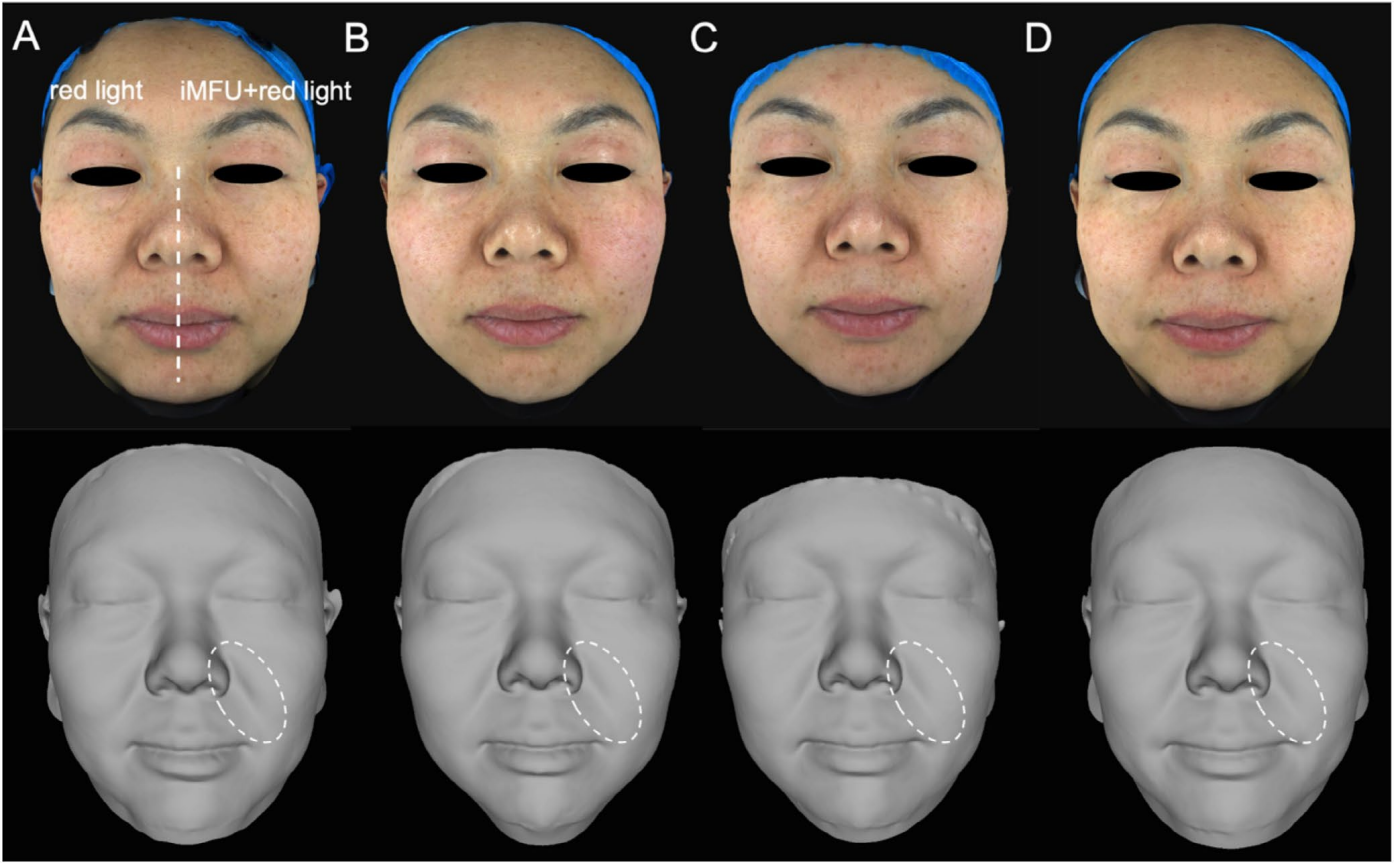
Representative case 1 (a 45‐year‐old female patient): 3D imaging photographs and grayscale images of treated side (iMFU+red light) and control side (red light) before treatment (A), immediately after treatment (B) and 1 month (C) and 2 months (D) after treatment.

**FIGURE 5:**
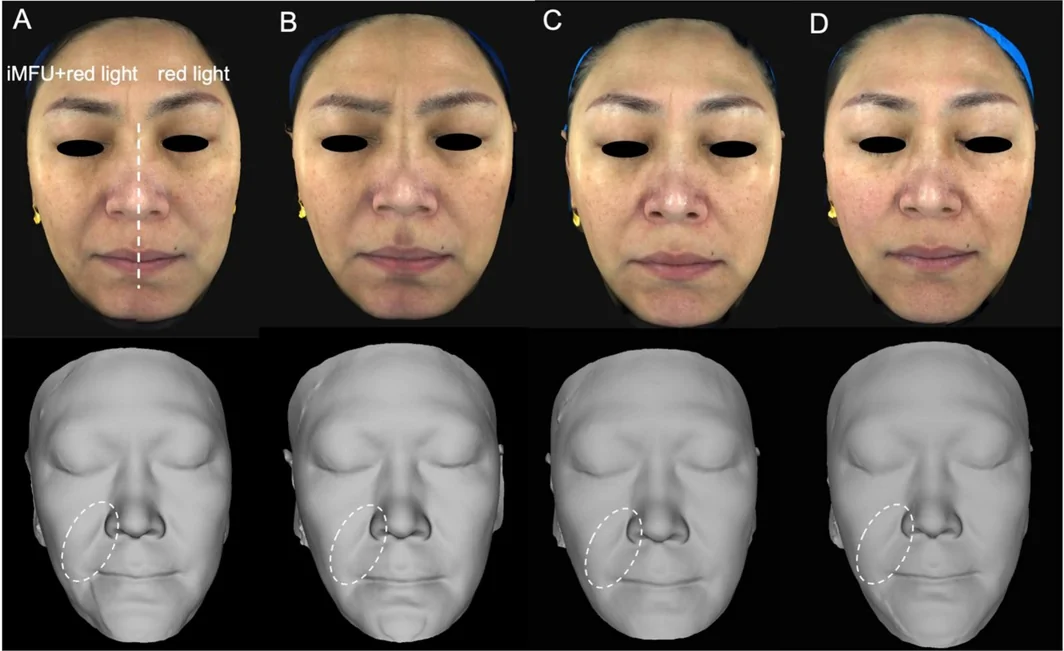
Representative case 2 (a 47‐year‐old female patient): 3D imaging photographs and grayscale images of treated side (iMFU+red light) and control side (red light) before treatment (A), immediately after treatment (B) and 1 month (C) and 2 months (D) after treatment.

In this prospective, split‐face study, we compared the effect of iMFU treatment on the improvement of nasolabial folds and perioral wrinkles. Representative photographs acquired through facial 3D imaging system at baseline, immediately after treatment and 1 and 2 months after treatment were presented in Figures 4 and 5. Participants exhibited characteristic manifestations of skin aging, encompassing cutaneous laxity, profound wrinkles, unclear and asymmetrical facial contours on both sides of face initially. Visual observation revealed that after iMFU treatment, the facial contours became clear, and the phenomena of facial sagging was significantly improved.

**FIGURE 6:**
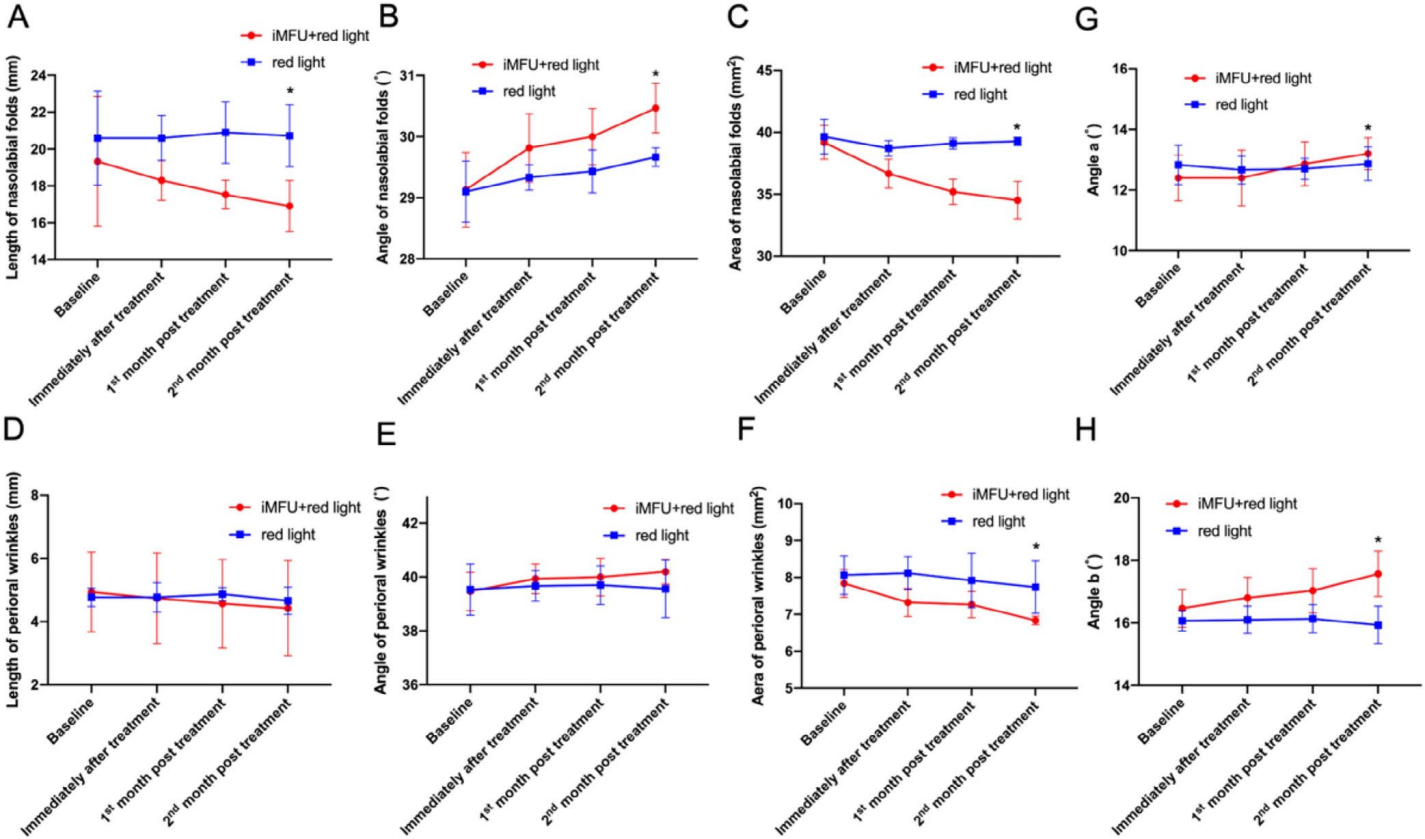
Variations of the length, area and angle of nasolabial folds and perioral wrinkles, and angles a and b before treatment, immediately after treatment and 1 and 2 months after treatment.

Quantitative morphometric analysis at the 2‐month follow‐up interval demonstrated post‐intervention reductions in nasolabial folds parameters on iMFU sides, with mean decreases of 18.5% in linear length (*p* < 0.01), 3.6% in angular deviation (*p* = 0.011), and 12.3% in surface area (*p* = 0.02) within treatment fields (Figure 6A–C). Perioral wrinkles exhibited analogous dimensional improvements, showing 7.2% length contraction, 3.1% angular correction, and 10.6% area reduction. Although the anti‐aging effect can be clearly observed immediately after the treatment, longitudinal assessment revealed progressive optimization, with peak efficacy manifesting at the 2‐month follow‐up interval, reflecting the enhanced lifting effect with the extension of time, as shown in Figure 6D–F. However, during the twice follow‐up processes, the angle, length, and area of perioral wrinkles and nasolabial folds in control sides showed no significant improvement. Quantitative analysis revealed obvious changes following iMFU treatment: side face angle a demonstrated a significant increase from 12° ± 2.6° to 13° ± 1.5°, while the mandibular angle b similarly increased from 16° ± 2.4° to 18° ± 1.9°. Notably, quantitative value of angle a and b on control sides remained statistically unchanged (Figure 6G,H).

### 3.2 Secondary Outcomes

**FIGURE 7:**
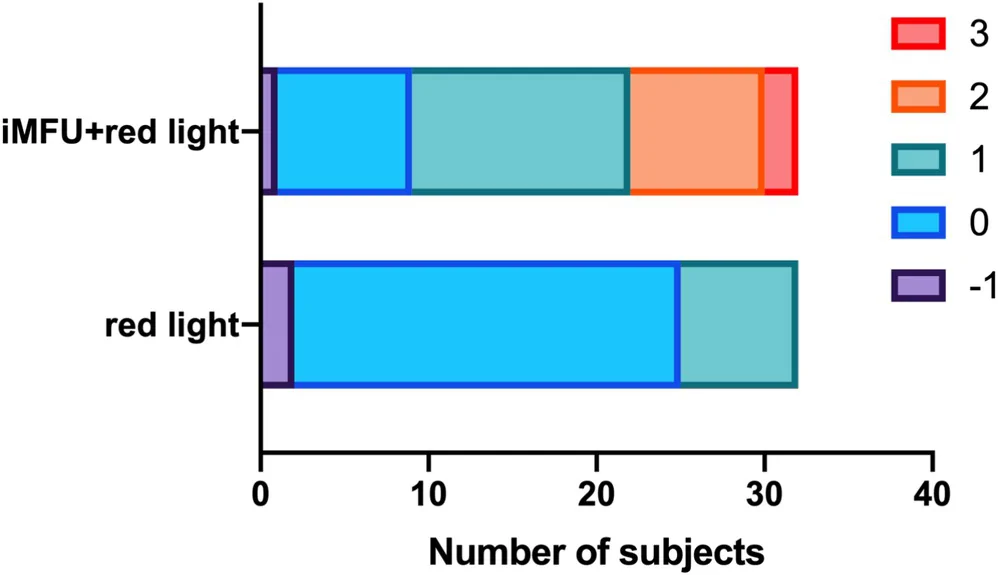
GAIS scores of combined sides and control sides, respectively, 2 months after treatment.

Quantitative assessment via GAIS demonstrated significant enhancement in iMFU sides at the 2‐month follow‐up interval (Figure 7). 81.3% of participants (*n* = 26/32) exhibited measurable improvement in photoaging manifestations, with 71.9% (*n* = 23/32) achieving clinically significant improvement (≥ 1‐grade change). Conversely, the control sides showed markedly reduced efficacy, with only 21.9% of subjects (*n* = 7/32) exhibiting any detectable amelioration of aging characteristics.

**FIGURE 8:**
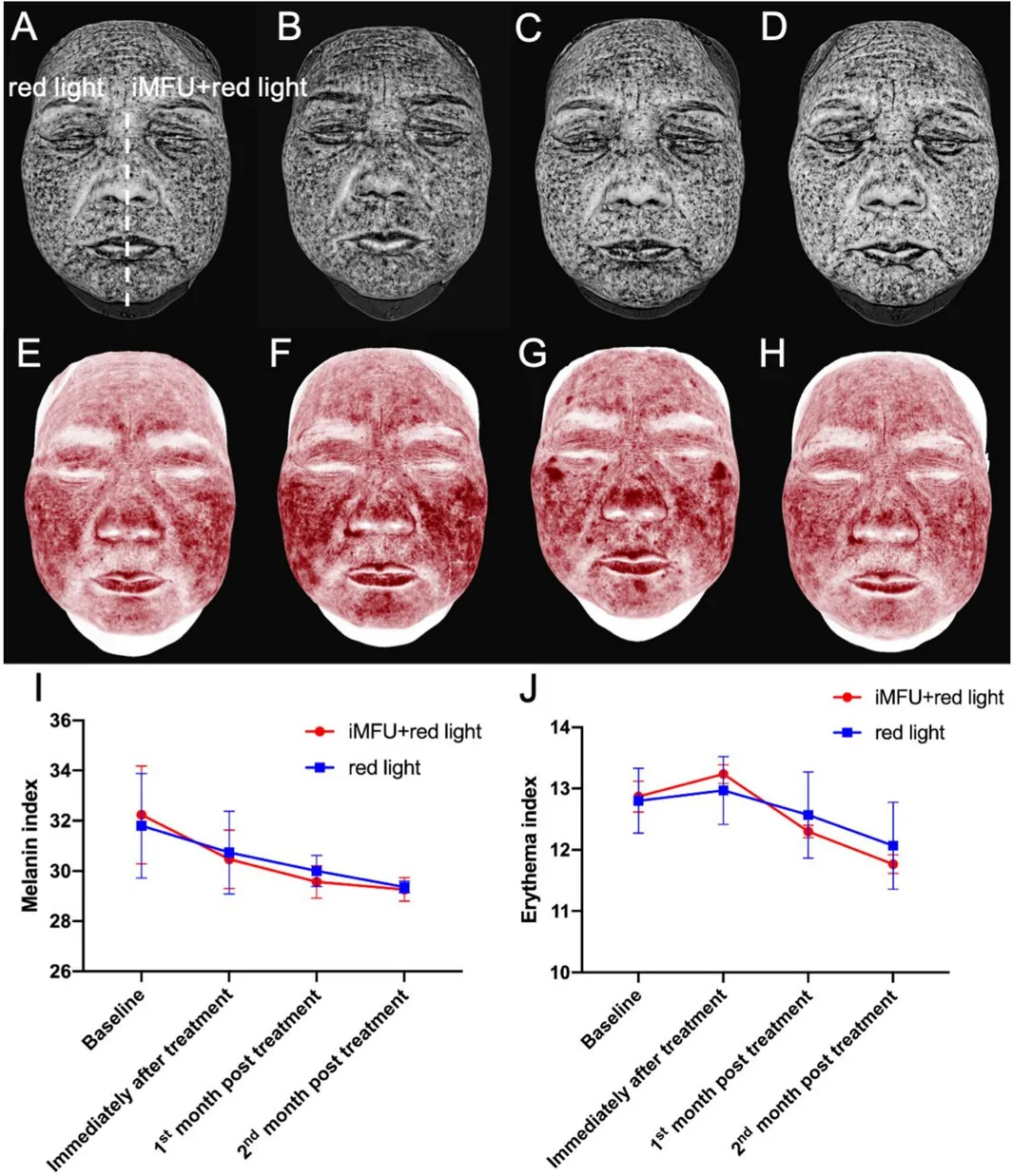
Representative case (a 63‐year‐old female patient): Melanin photographs of treated side (iMFU+red light) and control side (red light) before treatment (A), immediately after treatment (B) and 1 month (C) and 2 months (D) after treatment. Erythema photographs of treated side (iMFU+red light) and control side (red light) before treatment (E), immediately after treatment (F) and 1 month (G) and 2 months (H) after treatment. Melanin index (MI) (I) and erythema index (EI) (J) variations on treated sides and control sides before treatment, immediately after treatment and 1 and 2 months after treatment.

The reduction in MI and EI reflected significant improvements in skin color on both the combined and control sides. MI and EI in iMFU sides were significantly reduced at the 1st month and 2nd month follow‐up, respectively (Figure 8), and the differences persisted until the 2nd month follow‐up. Our observations indicated that iMFU induced transient facial erythema and edema immediately after treatment, resulting in a measurable but statistically insignificant elevation in EI values. However, these acute inflammatory responses resolved spontaneously, with all cutaneous parameters returning to pretreatment baselines within the 24‐h postoperative window. Surprisingly, the reduction of MI and EI was also statistically significant in control sides. In summary, the skin color of both sides improved; however, the improvement was more obvious on the combined side.

### 3.3 Subjects' Satisfaction and Side Effects

**FIGURE 9:**
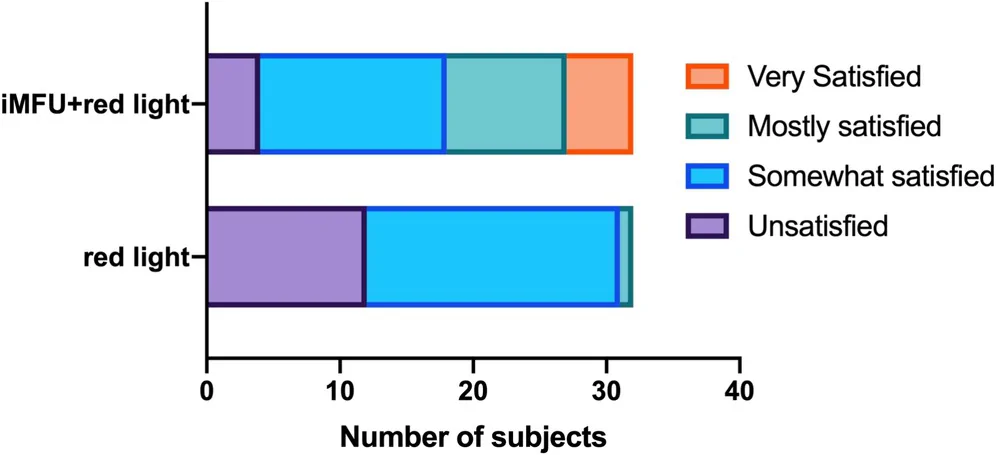
Subjects' satisfaction scores on combined sides and control sides.

Subjects' satisfaction between the split sides showed significant differences and the overall patients' satisfaction rate on iMFU sides was 75% (Figure 9).

The predominant adverse events associated with iMFU treatment comprised transient erythema (incidence: 46.9%), edema (6.2%), and procedural pain. Quantification via VAS revealed marginally elevated pain during iMFU application (mean score 2.4 ± 0.8). Treatment‐associated discomfort manifested primarily as distending pain (35% of subjects) and tingling pain (30%), with less frequent reports of neuropathic‐type sensations including electric shock (8%) or thermal discomfort (5%). Erythema and edema usually subside within a few hours after treatment. Intermittent cold application can effectively relieve discomfort caused by erythema and swelling. In addition, one patient developed toothache within 1 week of treatment and gradually resolved 1 month after treatment.

## 4 Discussion

Skin aging is divided into two types: exogenous aging, also known as photoaging, and endogenous aging. While minimizing ultraviolet exposure can prevent exogenous aging, endogenous skin aging remains largely unavoidable [2, 23]. Skin aging encompasses both photoaging and inevitable endogenous aging, the latter manifesting as collagen depletion, fascial weakening, and progressive facial contour loss. This multicenter, prospective, split‐face study evaluated a novel intelligent MFU (iMFU) system for facial rejuvenation. iMFU produced significant improvements in nasolabial fold dimensions (18.5% length reduction, *p* < 0.01), perioral wrinkle parameters (7.2% length reduction, *p* < 0.001), facial contour angles, and pigmentation indices within 2 months, with a favorable safety profile.

The biomechanical mechanism of MFU involves thermal denaturation and contraction of collagen fibers in subcutaneous adipose tissue, primarily through disruption of intermolecular hydrogen bonds. This molecular‐level alteration induces structural reorganization of collagen chains into shorter, more stable configurations. Subsequently, neocollagenesis initiates around these thermal coagulation zones, generating progressive tissue remodeling that produces clinically observable lifting and tightening effects on lax skin [24, 25, 26].

MFU delivers focused ultrasonic energy to the SMAS, generating thermal coagulation zones that induce immediate collagen contraction and subsequent neocollagenesis over weeks to months [25, 27]. The iMFU device employs four transducer configurations: adaptive variable focal‐length sensors (T ~ and S ~ probes) adjusting depth from 3.5 to 5.5 mm under real‐time ultrasound guidance, and fixed 3.0 mm sensors (T3.0 and S3.0) targeting the superficial dermis. This adaptive technology enables individualized depth calibration based on regional SMAS topography. However, as no head‐to‐head comparison with conventional MFU platforms was performed, no superiority claims can be made.

Our clinical evaluation revealed differential therapeutic outcomes based on facial adipose volume. Subjects with higher adipocyte density demonstrated significant improvement in gravitational ptosis and contour definition following focused ultrasound treatment, attributable to collagen remodeling within the superficial musculoaponeurotic system. Conversely, patients with lower subcutaneous fat exhibited notable reduction in wrinkles through dermal condensation effects. Similarly, younger participants exhibited enhanced therapeutic responses relative to older individuals. This differential efficacy may be attributable to iMFU's targeting of SMAS layer coupled with its integrated real‐time tissue impedance monitoring. This proprietary technology enables dynamic depth adjustment calibrated to individual anatomical variations across facial regions. Therefore, the subject' satisfaction rate is relatively high among patients with higher facial adipose volume.

The results showed that there was a statistically significant difference in the GAIS score between the treated side and the control side of the subjects. The variable focal length depth sensors used in this study customized the treatment depth for the subjects based on different facial regions of the subjects, and conducted precise treatment at a depth of 3.5–5.5 mm in the significantly sagging areas. Targeted fat decomposition treatment at a depth of 3.0 mm is carried out in areas rich in facial fat, aiming to achieve a harmonious improvement in facial aesthetics. Furthermore, we found that there was a significant improvement in facial contours immediately after iMFU treatment. The improvement in facial aging was the most significant 1 month after the treatment, and there was still a significant improvement at the end of the 2‐month follow‐up.

MI and EI, another representative characteristic of photoaging, represent the range and degree of erythema and melanin, respectively [2]. This investigation revealed that concomitant with significant facial contour enhancement, the combined intervention elicited quantifiable reductions in erythema index and melanin density within treatment zones. EI on both sides of the face showed a slight increase immediately after treatment, which might be related to the transient stimulation response associated with the red‐light illumination and iMFU treatment. Joyce et al. have reported that MFU can improve UV‐induced pigmentation and stimulate neocollagenesis and neoelastogenesis, presenting as a safe and effective treatment option for melasma [20, 28]. Notably, analogous cutaneous improvements manifested in control fields, suggesting the specific biological effects of 633 nm red light on intracellular chromophores and mitochondria, demonstrating therapeutic benefits including accelerated wound healing, anti‐inflammatory effects, relief of muscular fatigue, and enhanced tissue repair [22, 29].

A key limitation of this study is the 2‐month maximum follow‐up duration, which is insufficient to fully characterize the durability of iMFU‐mediated effects, as collagen remodeling following thermal injury typically progresses over a 3–6 month period. Further studies with follow‐up extending to at least 6 months are warranted to evaluate the long‐term efficacy and sustainability of outcomes.

## 5 Conclusion

This investigation systematically evaluated the safety and therapeutic efficacy of iMFU for mid‐to‐lower facial rejuvenation. IMFU demonstrates clinical viability, exhibiting significant effects in skin tightening and tone improvement, with promising clinical applicability. Further validation with larger sample size and extended longitudinal assessment is warranted to establish sustained therapeutic strategy.

## Author Contributions

S.S. performed the research and wrote the paper. T.X., X.L.W., and P.W. designed the research study. J.L., Q.F., M.W., and X.L. contributed essential reagents or tools. S.G., C.C.W., L.M., and Q.Y. analyzed the data. P.W. revised the manuscript.

## Funding

This work was supported by the 2024 Research Project Plan of Chinese Association of Rehabilitation Medicine (KFKT‐2024‐KY‐021), 2025 Annual Tongji University “Medicine + X” Interdisciplinary Research Project, and the Investigator‐Initiated Trial Fund of Shanghai Skin Disease Hospital (20240731099).

## Consent

Written informed consent to publish clinical photographs was obtained from all participants prior to inclusion in this study.

## Conflicts of Interest

The authors declare no conflicts of interest.

## Data Availability

The data that support the findings of this study are available on request from the corresponding author. The data are not publicly available due to privacy or ethical restrictions.
